# Supplementary figures and images for: An optimized pipeline for live imaging whole Arabidopsis leaves at cellular resolution
Source: Plant Methods. 2023 Feb 1;19:10. doi: 10.1186/s13007-023-00987-2 (PMC9890716; doi:10.1186/s13007-023-00987-2)

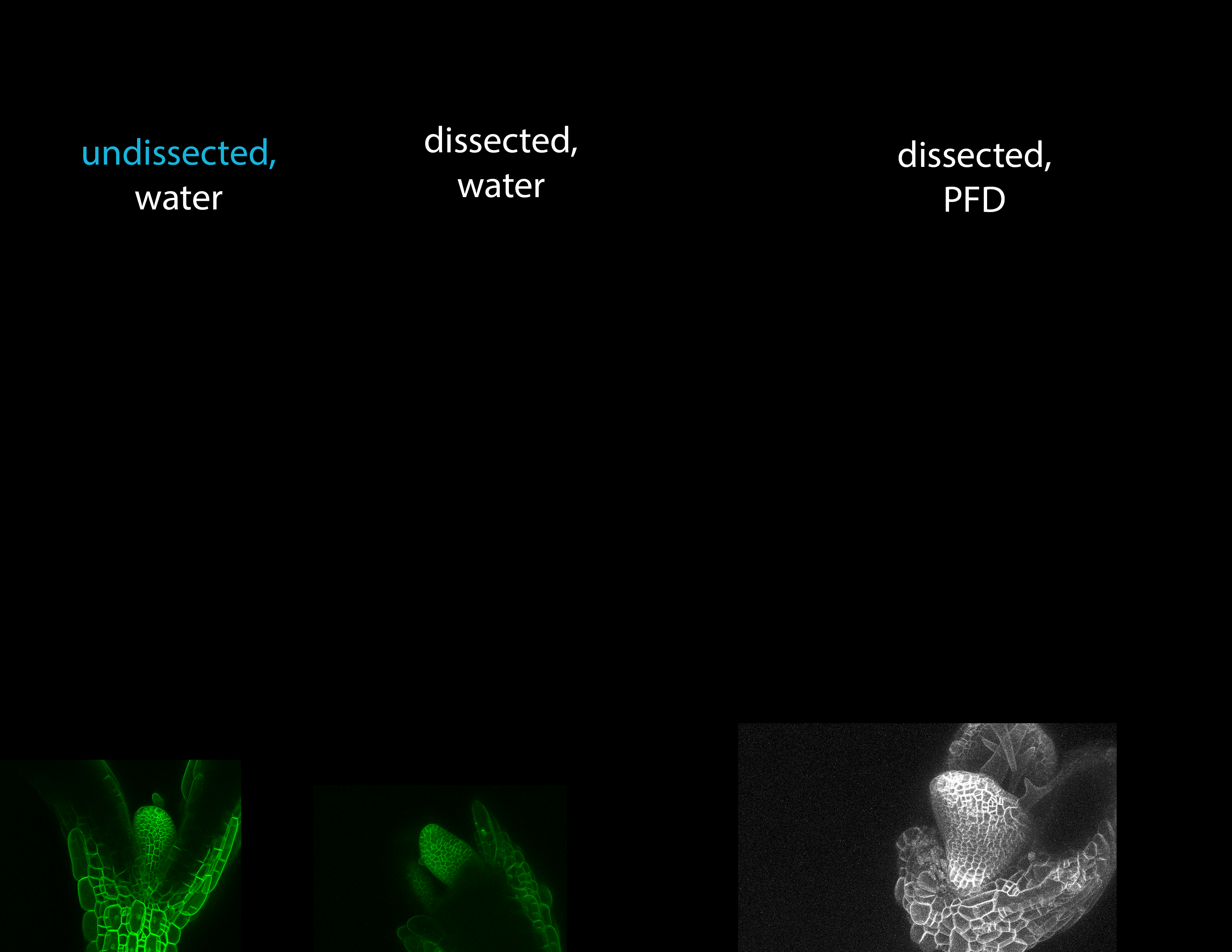

Supplement: Supplementary file 3 — Additional file 3. Video S3. Perfluorodecalin mounting solution improves sample vitality over waterbased solutions. Animation of undissected and water submersed or dissected and water submersed or dissected and perfluorodecalin submersed samples (left to right). The growth of the first two samples begins to slow and eventually stalls from 5 to 7 DAS. The perfluorodecalin sample continues to grow. Maximum intensity projections of confocal stacks are shown false colored in green or gray. Red scale bar = 100 µm. [file 13007_2023_987_MOESM3_ESM.gif]
