# Supplementary material for: An optimized pipeline for live imaging whole Arabidopsis leaves at cellular resolution
Source: Plant Methods. 2023 Feb 1;19:10. doi: 10.1186/s13007-023-00987-2 (PMC9890716; doi:10.1186/s13007-023-00987-2)
Supplement: Supplementary file 6 — Additional file 6. Supplemental_code.zip The supplemental code file contains scripts to cary out the analysis. batchtiff.py is a script to convert .lsm files to .tiff files recursively through a directory. Multi_resize.py is a MGX script to resize voxels. 2021_mesh_creation_mgx3.task is a MGX task list with processes for making meshes. 2021_parent_correcting.task is a MGX task list for correcting parent labeling of meshes. Annotated_task_list.docx is a list of MGX processes with parameter values and notes. Iterative_growth_and_measures.py is a MGX script to apply various cellular measures and produce snapshots of heatmaps. li_preprocess.R is an R script used to preprocess data. li_plotting.R is an R script used to plot the data. light_paper_plots.R is an R script containing plot formatting. [file 13007_2023_987_MOESM6_ESM.zip › supplemental_code/annotated_task_list.docx]

**Creating meshes:**

*Stack/MultiStack/Copy Main to Work Stack*

*Stack/MultiStack/Copy Work to Main Stack*

These processes are used in tandem to edit confocal stacks in the ‘Work’ stack with the voxel edit tool. Voxels can only be edited out in the ‘Work’ stack. Stacks are copied back and forth until all undesired confocal signal is removed. The final edited stack should be saved from the ‘Main’. Merged stacks will also be saved to the active stack’s ‘Work’ and should use *Copy Work to Main Stack* to be saved from ‘Main’.

*Stack/MultiStack/Merge Stacks*

Method: max

Interpolation: Linear

This process merges two stacks when they are loaded in each ‘Main’ stack. With these parameters, it takes the maximum intensity voxel of the two stacks, this helps with image quality.

*Stack/CImage/Gaussian Blur*

Radius: 5

This process blurs the main stack to help with creating a smooth mask. This radius worked well for most leaf samples without taking too long to run.

*Stack/Morphology/Edge Detect*

Threshold: 5000

Multiplier: 2.0

Adapt Factor: 0.3

Fill Value: 30000

This process creates the mask of the blurred stack. The threshold should be set based on the signal from the confocal stacks. For leaf samples, we found lowering it to 5000 was helpful. The rest of the parameters determine how signal is filled in as a mask. We kept them at default values.

*Stack/Morphology/Closing*

X Radius: 5

Y Radius: 10

Z Radius: 3

Round Nhbd: No

This process was used for meshes where the mask had holes. This usually happened in later time points where large cells had no signal in the center. The radii should be set to about the size of the holes. The clipping planes and scale in the corner can be used to help determine the size.

*Mesh/Creation/Marching Cubes Surface*

Cube size (µm): 5.0

Threshold: 5000

This process fits a surface of cubes to the mask that was created. 5 µm is a sufficient cube size for a coarse mesh of leaves, but smaller samples that are more curved may need smaller cube sizes. The threshold is the signal level at which cubes will be made.

*Mesh/Selection/Select Bad Normals*

After selecting and deleting regions of the mesh that do not have signal, sometimes weird shapes or very tall maxima will be left in the mesh. This process finds these peaks and selects them so they can be deleted.

*ProcessName: Mesh/Structure/Subdivide*

This process subdivides the triangles that make up the mesh. For large samples, only run this once before attempting to project the stack signal and segment cells to save RAM space.

*Mesh/Structure/Smooth Mesh*

Passes: 20

Walls Only: No

This process evens the spacing between the nodes and edges that comprise the triangles in a mesh. 20 passes is good for larger and generally flat samples like leaves where small differences in marker signal should be smoothed over. After running this process, it is good to take a cross section of the surface and stack with the clipping plane. This will allow you to check that they follow each other closely.

*Mesh/Structure/Shrink Mesh*

Distance(µm): 5

This process may be necessary if the surface is too high above (or below) the stack. The distance should be an estimate of the distance of the surface from the stack. Negative values can also be supplied to expand the mesh out to a surface. The mesh should be resmoothed after running this process

*Mesh/Signal/Project Signal*

Use absolute: No

Min Dist (µm): 2.0

Max Dist (µm): 6.0

Min Signal: 0.0

Max Signal: 60000.0

This process dictates the projection of the stack onto the mesh. It should be run a few different times changing the min and max dist parameters until the signal of the stack seems well-represented. The clipping planes can be used to take a cross section of the mesh over the stack and used with the scale bar on the side to estimate the range to use. The min and max signal can be used to limit what levels of signal will be projected, but we kept them at defaults. This process can be run repeatedly with the four following processes to gradually refine the mesh segmentation.

*Mesh/Segmentation/Auto Segmentation*

Update: Yes

Normalize: Yes

Blur Cell Radius (µm): 5

Auto-Seed Radius (µm): 5

Blur Borders Radius (µm): 1

Normalization Radius (µm): 45

Border Distance (µm): 1.0

Combine Threshold: 1.15

This process automatically seeds and segments cells. Setting update to ‘yes’ will display each step of segmentation on the mesh which can help with setting parameters. Blur Cell and Auto-seed radius should be the size of the smallest cells (per MGX documentation). For leaves we found they should be set a little higher. Setting to the smallest size tends to over-segment. Blur borders radius and border distance should be about the width of the cell borders (per MGX documentation). Normalization radius should be estimated from the largest cells until about 100 µm in later time points. Setting this too high will under-segment cells. Normalization and Auto-Seed/Blur Cell radii parameters values usually need to be balanced to properly segment most medium-sized cells and assume that the largest cells will need to be combined and the smallest cells will need to be segmented apart. Combine threshold is an estimate of the signal of the border versus the inside signal of a cell. It tries to estimate what cells are over-segmented and combine them. Setting it higher can over-combine cells.

*Mesh/Structure/Subdivide Adaptive Near Borders*

Max Area(µm²): 0.1

Border Dist(µm): 2.5

The Max Area is the area below which a triangle in the mesh won’t be subdivided. We usually set this very small so most triangles will be subdivided. The Border Dist is the maximum distance a triangle can be from the border and still be subdivided. We found these values worked well to keep meshes small, but prevent four way junctions between cells. Regular subdivide can be used if meshes are for small samples and RAM availability is not an issue.

*Mesh/Structure/Smooth Mesh*

Passes: 1

Walls Only: No

This process should be run every time a subdivision is made to keep the spacing between triangles relatively even.

*Mesh/Segmentation/Segmentation Clear*

If the mesh is still coarse and the segmented borders do not appear smooth over the cell membrane signal, then this process should be run and the preceding four processes repeated. Repeating this cycle three times usually gives nicely refined cell borders.

*Mesh/Cell Mesh/Tools/Relabel Fragments*

Relabel Threshold: -1

This process will unlabel any cells that have the same label, but are not contiguous in the mesh. The same label can be found at two places in a mesh if the watershed segmentation goes awry. This should be run after a reasonable segmentation is achieved with the above four processes.

*Mesh/Segmentation/Watershed Segmentation*

Steps: 50000

This process may need to be run if Relabel Fragments deletes labels from the mesh and/or new cells are seeded in refining the segmentation. Using the lasso select or square select can be a good idea to limit this process only to the part of the mesh where it is needed to make the process run faster. Setting steps higher will update the screen less frequently and make the process run faster.

*Mesh/Cell Mesh/Fix Corner Triangles*

This process should be run on meshes with a relatively final segmentation. Sometimes it will crash MGX, so the mesh should always be saved before running this process. The process will find any regions in the mesh where corner triangles lead to weird cell borders. This process must pass before check correspondence will pass (see Parent Correcting section below).

*Mesh/Selection/Extend by Connectivity*

*Mesh/Selection/Invert Selection*

These processes should be used after deleting any part of the mesh. Disconnected regions can result. The main mesh should be selected, then these processes run together. Whatever is selected after running these processes is a disconnected region that should be deleted.

**Parent correcting:**

*Mesh/Selection/Select Bad Normals*

This may need to be run again if areas of the mesh are deleted after running this initially.

*Mesh/Cell Mesh/Fix Corner Triangles*

This needs to be run again after changing segmentations and before running Check correspondence

*ProcessName: Mesh/Selection/Select Unlabeled*

Replace selection: No

This process is helpful to highlight where labels have been deleted, for example, after running Relabel fragments.

*Mesh/Deformation/Set Correct Parents*

*Mesh/Deformation/Mesh 2D/Auto Parent Labelling 2D*

Max Steps: 10

Auto Fix Parents Depth: -1

Retreat %: 0

These processes can be run together to automatically label parents. Default parameters were always used. Samples usually achieved between 10-40% labeling in a first pass. The main issues with label progression were cells where divisions were ambiguous or there were four-way junctions that would need to be corrected anyway. Over time we found this was no faster than manually labeling. Because while manually labeling, it is also possible to look out for four-way junctions and subdivide and re-segment these along the way.

*Mesh/Cell Axis/PDG/Check Correspondence*

Show correspondence: No

Convert to Cell Mesh: No

New: No

This process checks that all the cell labels in the first time point touch the same borders when they are tracked as parent labels in the second time point. Show correspondence draws lines between corresponding junctions. This may be helpful in finding errors. Convert to Cell Mesh simplifies the borders to just the lines necessary to connect junctions. This simplification makes it harder to have landmarks and figure out junction errors.

*Mesh/Structure/Subdivide*

*Mesh/Structure/Smooth Mesh*

Passes: 1

Walls Only: No

*Mesh/Segmentation/Segmentation Clear*

*Mesh/Segmentation/Watershed Segmentation*

Steps: 50000

These processes can be run in order to increase the density of triangles at four-way junctions and re-segment to resolve the conflicts detected by check correspondence. The square or lasso selection tools can be used to select only a small area around the four-way junction.

*Mesh/Segmentation/Label Selected Vertices*

Label: 0

This process can be used to change the label of selected vertices. Label should be used to designate which label to add. Using the eye dropper tool will make any cell’s label appear in the bottom left. This value can then be entered. Label 0 will clear the labels.

*ProcessName: Mesh/Cell Mesh/Tools/Relabel Fragments*

Relabel Threshold: -1

This process may need to be run again to help resolve errors detected by check correspondence.

*Mesh/Selection/Extend to Whole Cells*

Segmentation proceeds by watershed (coloring from a seeded point until another color is run into). This means that edges of the mesh also need to be designated with one border color. Sometimes auto-segmentation will also over-segment the edges. If these colors are not deleted, the values measured for these “cells” will be included in the attribute map and any measurements made subsequently. So, these “cells” should be deleted before analyzing data exported from the attribute maps. This process can be used with the lasso or rectangle selection tools to select cells at the mesh edge. The edge of the mesh segmentation can then be deleted with Clear segmentation.

*Mesh/Selection/Extend by Connectivity*

*Mesh/Selection/Invert Selection*

*Mesh/Selection/Select All*

As before, these processes can be used together with the lasso or rectangle selection tool to select the main connected mesh, then detect and rectify disconnected regions of the mesh.

*ProcessName: Mesh/Cell Axis/PDG/Compute Growth Directions*

This process is run once check correspondence passes to make sure that growth directions can be computed.

*Mesh/Selection/Select Labels*

Labels: 38010

Replace Selection: No

This process can be used to select any label or a list of labels in a mesh. It can be helpful to highlight cells that have weird or outlier behavior after data analysis.
